# Manchester Royal Infirmary

**Published:** 1895-03-16

**Authors:** 


### MANCHESTER ROYAL INFIRMARY.

Once again in the history of this institution the question of increased accommodation presents itself for decision to the governors. A special meeting of the trustees was held on the 26th ult. to consider the desirability of enlarging the Manchester Royal Infirmary, by one of the two schemes referred to in the annual report of the Board of Management. These were (*a*) to throw out two wings where porches now stand, *i.e.*, one in Portland Street and the other in George Street. These wings would occupy together about one-eleventh of the 14,735 square yards of the open area which remains, and would provide about one hundred more beds. (b) To raise the whole structure by one storey, and to lengthen the Broad Street wing and out-patients' department towards Parker Street, including the addition of an extra storey to the Nurses' Home, and the erection of a new mortuary and post-mortem room. In order to carry out either of these proposals it would be first necessary for the trustees to rescind their resolution of July 22nd, 1892, which forbids the erection of any further buildings upon the limited uncovered space still available. The committee favoured the first of these two proposals, but the meeting of trustees rejected both in favour of a proposal to provide the increased accommodation required by gradually rebuilding the infirmary on the present site. As this latter proposal was carried by a large majority, although a poll has been demanded by those who favour the first scheme, it is fair to assume that it will be adopted.

It will be apparent to the uninitiated that things have almost come to a deadlock in Manchester, so far as hospital building is concerned. The existing buildings known as the Royal Infirmary were condemned as insanitary many years ago by the late Mr. Netton Ratcliffe. The present hospital is designed upon a bad plan ill-adapted to the climate of this country —one which fails to permit thorough ventilation, and so renders the treatment of surgical cases difficult, not to say dangerous, despite the utmost skill on the part of the medical staff. Most independent people who have a knowledge of Manchester and the present infirmary site, as well as of the condition of the existing buildings, have been unanimous for years in declaring that the proper course, in the interests of the poor population of Manchester, is to select another site, and there to remove the hospital from the centre of a crowded city like Manchester, where the elements which make for recovery from disease are largely neutralised by the impurities of the atmosphere and the restricted character of the site. Mr. Brookbank, who proposed to rebuild the present infirmary on the present site, failed to mention this proposal, which is undoubtedly the best of all. He did, indeed, allude to a proposal to erect a hospital elsewhere than on the present site, a portion of which was to be then cleared and let off for building purposes, or sold to the Corporation as an open space. Upon the remaining site the infirmary would conduct a central hospital for accidents, out-patients, and emergency cases. If this proposal were adopted the trustees would have to maintain two hospitals instead of one, at considerable extra expense and an increase of work for the staff. On the other hand, Mr. Brookbank maintained that the gradual reconstruction of the infirmary upon its present site, at the outside, would cost £75,000. This new infirmary would contain 160 additional beds, besides new lecture rooms and other accommodation, whilst the work of the hospital could proceed during the rebuilding without a break. He admitted that his plan would encroach on the limited uncovered area to the extent of from 1,100 to 1,200 square yards, but the other wings would be taken down to the first story, and the portion adjoining the front block demolished altogether. The remaining wings of the old building would be reduced to one story and top-lighted, and would furnish theatres, lecture-hall, work-rooms, &c. The rebuilding could be carried out in three portions during several years, and the new central pavilion might be delayed until it was found absolutely necessary to erect it. It appears from the proceedings at the meeting that the medical staff, having considered all these schemes, are in favour of rebuilding the infirmary on the present site.

If the trustees, the profession, and the public of Manchester are determined, whatever happens, that their infirmary shall remain for ever on probably the worst site that can be found for it in the whole town, there is nothing more to be said. Assuming, then, that the Manchester Royal Infirmary is to be rebuilt on the present site, we must urge the trustees and the medical staff to secure that the whole of the soil on the existing site shall be excavated to a sufficient extent and replaced by fresh and pure soil brought from outside. It will be necessary for them further to insist, if the requirements of medical science and hygiene are to prevail, as they ought to prevail, that the design for rebuilding the hospital on its present site shall be subject to full criticism, whoever may prepare it, so as to secure that the evils and dangers of faulty construction shall be eliminated entirely. To insist upon putting up a new building costing many thousands of pounds—which means an expenditure of from £100,000 to £150,000 at least—on the present site, with all its disadvantages, and not to provide that the best plan shall be secured, so as to make the new buildings as perfect as possible before a single stone of them is laid, would be to commit an error more grievous than any which have been committed in the past history of this unfortunate building, and that is saying much.

